# A direct collocation framework for optimal control simulation of pedaling using OpenSim

**DOI:** 10.1371/journal.pone.0264346

**Published:** 2022-02-22

**Authors:** Sangsoo Park, Graham E. Caldwell, Brian R. Umberger

**Affiliations:** 1 Department of Kinesiology, University of Massachusetts Amherst, Amherst, Massachusetts, United States of America; 2 College of Medicine, Korea University, Seoul, South Korea; 3 School of Kinesiology, University of Michigan, Ann Arbor, Michigan, United States of America; North Carolina State University, UNITED STATES

## Abstract

The direct collocation (DC) method has shown low computational costs in solving optimization problems in human movements, but it has rarely been used for solving optimal control pedaling problems. Thus, the aim of this study was to develop a DC framework for optimal control simulation of human pedaling within the OpenSim modeling environment. A planar bicycle-rider model was developed in OpenSim. The DC method was formulated in MATLAB to solve an optimal control pedaling problem using a data tracking approach. Using the developed DC framework, the optimal control pedaling problem was successfully solved in 24 minutes to ten hours with different objective function weightings and number of nodes from two different initial conditions. The optimal solutions for equal objective function weightings were successful in terms of tracking, with the model simulated pedal angles and pedal forces within ±1 standard deviation of the experimental data. With these weightings, muscle tendon unit (MTU) excitation patterns generally matched with burst timings and shapes observed in the experimental EMG data. Tracking quality and MTU excitation patterns were changed little by selection of node density above 31, and the optimal solution quality was not affected by initial guess used. The proposed DC framework could easily be turned into a predictive simulation with other objective functions such as fastest pedaling rate. This flexible and computationally efficient framework should facilitate the use of optimal control methods to study the biomechanics, energetics, and control of human pedaling.

## Introduction

Optimal control theory paired with musculoskeletal modeling and simulation is a powerful approach for studying the biomechanics, energetics, and control of human movement [1–4]. In particular, musculoskeletal simulation has been used to study the neural control of pedaling as a representative locomotor task that has fewer stability requirements than walking [5–7]. The simulation approach has also been used to improve our understanding of the biomechanics and energetics of pedaling with applications that range from rehabilitation to athletic performance [8–11]. However, there are considerable challenges in applying optimal control theory to investigate the mechanics, energetics and control of human movement, such as the substantial computational cost, the complexity of developing musculoskeletal models, and until recently the lack of user-friendly simulation environments.

The computational cost of generating optimal control simulations of human movement has until recently been a major limiting factor to its widespread adoption. This limitation can largely be overcome by using the direct collocation (DC) optimal control framework, which exploits sparsity in the problem formulation to substantially lower the computational cost of simulating human movement compared with traditional methods [12–16]. Despite this advantage, the DC method has rarely been used to solve optimal control pedaling problems. Although Kaplan and Heegaard had already demonstrated the potential of DC for generating rapid simulations of human pedaling two decades ago [15], their complex symbolic formulation and lack of available models or code has likely been a factor limiting the adoption of their approach.

The obstacles to implementing computational models of the musculoskeletal system can be substantially reduced using software packages such as OpenSim, a freely available musculoskeletal modeling software that has seen increased use within the biomechanical modeling community [17,18]. OpenSim provides a graphical user interface in which users can create a musculoskeletal model and perform a variety of analyses including static optimization and forward simulation, without having to derive the dynamical equations of motion in symbolic form. Further, the models and analyses can be utilized in other programming environments via a robust application programming interface (API), allowing customized optimization problem formulation. One candidate for interfacing with OpenSim libraries is MATLAB (The MathWorks, Inc.), a common scripting language used in science and engineering that provides both native optimizers (e.g. *fmincon*) and an interface (i.e., MEX) to access external optimization solvers such as IPOPT [19]. The OpenSim-MATLAB interface has been used to successfully solve DC optimal control problems of vertical jumping [12], walking [14,20], and running [14], but to our knowledge has not be used for simulating pedaling.

Thus, the aim of this study was to develop a DC framework for optimal control simulation of human pedaling within the OpenSim modeling environment. First, we developed a planar musculoskeletal model of the lower limbs and bicycle crank arm in OpenSim. Using the OpenSim API, the DC method was formulated in MATLAB (The MathWorks, Inc.) to solve optimal control pedaling problems that track a set of experimental human pedaling data. The quality of the optimal solution was evaluated by comparing simulation results with the experimental data. To facilitate other scientists being able to leverage our work in their own research, the model and sample codes are freely available at the GitHub repository (http://github.com/sangsoopark1739/pedaling_DC_OpenSim).

## Method

### Bicycle-rider model

**Fig 1:**
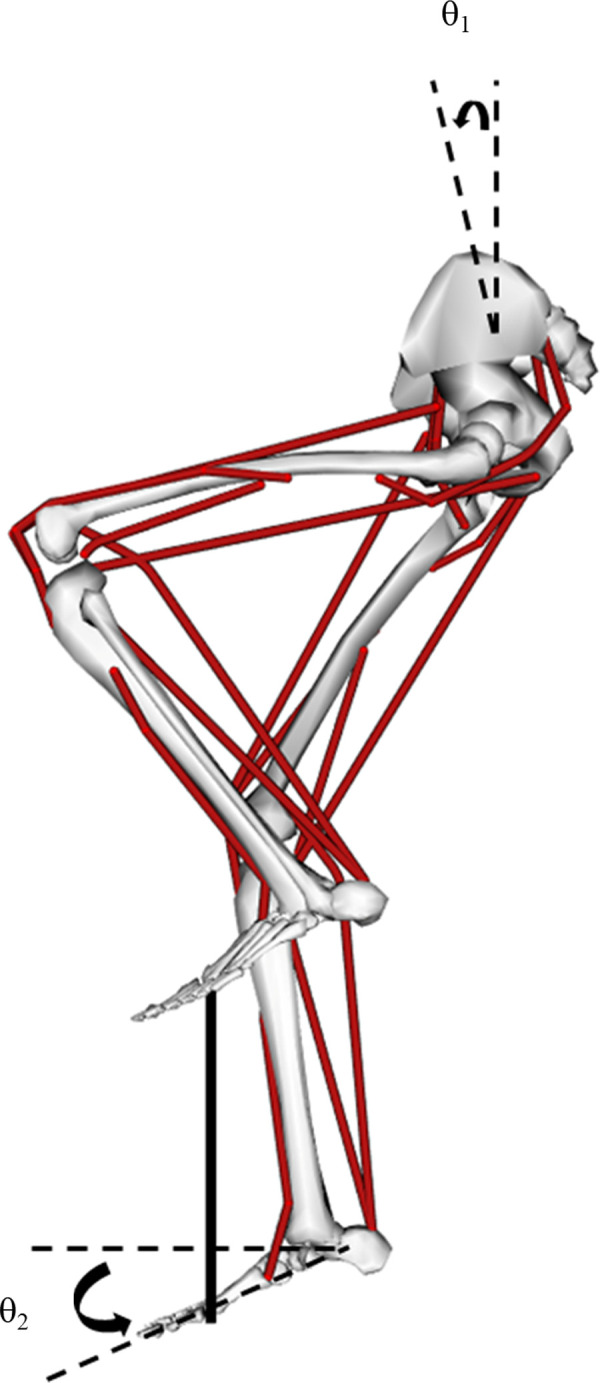
The bicycle-rider model in OpenSim. The developed 2-D bicycle-rider model consists of eight rigid segments (bicycle crank, pelvis, paired thighs, lower legs, and feet) and nine muscle tendon unit per leg (iliopsoas, gluteus maximus, vasti, rectus femoris, hamstrings, biceps femoris short head, gastrocnemius, soleus, and tibialis anterior). Pelvic tilt angle (θ_1_) was set to 11.5° and pedal angle (θ_2_) was determined by angular deviation between the horizontal and line connecting from the toe to the heel.

A two-dimensional (2-D) bicycle-rider model was developed in OpenSim [17] to solve optimal control pedaling problems using the DC method [13,21]. The model consists of eight rigid segments and nine muscle tendon units (MTUs) per leg (Fig 1), based on a recent model developed to simulate movements with large leg flexion angles such as occur in pedaling [22]. The rigid segments represent the bicycle crank, pelvis and paired thighs, lower legs, and feet. The hip, knee and ankle joints were modeled by hinge joints with coupled 2-D translation for the knee joint center of rotation [23]. The rotational axis of the crank was also modeled as a hinge joint. The pelvis segment center and rotational axis of the crank were fixed in space, representing seated pedaling on a stationary ergometer. Stiff springs (‘*PointToPointSpring*’ function in OpenSim, spring constant, k = 100,000) were used to connect the foot segments to the crank segment at a location representing the distal end of the metatarsals, creating a closed kinematics chain with ‘soft’ constraints between the feet and crank segments [24]. In the model, pedal angles were defined as the angular deviation from the horizontal of a straight line connecting the toe to the heel (Fig 1). Positive pedal angles in the model represent the toe being below a horizontal line representing a pedal angle of zero (0°). The mean pelvis angle across all participants was used in this model (11.5°; Fig 1), though there was a difference between males (9.3 ± 6.8°) and females (17.8 ± 4.9°), on average. Because the model and code are made available, interested readers could test the effects of these differences in pelvis angle for themselves. Details for the experimental data are described in a later section.

Each leg was actuated by nine muscle tendon units (MTUs): iliopsoas (IP), gluteus maximus (GMAX), vasti (VAS), rectus femoris (RF), hamstrings (HAM), biceps femoris short head (BFsh), gastrocnemius (GAS), soleus (SOL), and tibialis anterior (TA). A three-component equilibrium muscle model was used to represent each MTU, due to its computational efficiency and ability to match experimental data [25]. The force-producing potential of each MTU was determined by the force-velocity-length (F-V-L) relation of the contractile component (CC), and separate force-extension (F-Ext) relationships of series (SEC) and parallel (PEC) elastic components. Muscle-specific model parameters were selected to match net joint moments [26] and passive joint moments [27] in human participants. More details about the model development can be found in the Supplemental material 1 (S1 File).

The resistive torque applied to the bicycle crank varied inversely with crank speed at a constant power output. We estimated this resistive crank torque profile (*T*_*resistive*_) using the experimental crank torque and acceleration patterns.

$$T_{crank}-T_{resistive}=I_{eff}\times \alpha_{crank}$$ (1)

where *T*_*crank*_, *α*_*crank*_, and *I*_*eff*_ are mean crank torque, crank angular acceleration, and moment of inertia [28]. The effective crank moment of inertia (*I*_*eff*_) was 23.53 kg·m^2^ based on a gear ratio of 42/28 [28]. The resistive crank torque was smoothed and interpolated to 21 equally-spaced data points (*‘PrescribedForce’* function in OpenSim), which was applied about the rotational axis of the crank segment.

### Experimental data

Experimental pedaling data for the tracking optimizations were drawn from a previous study [29], including left pedal angles and pedal forces, and the activation patterns of ten left leg muscles. Data were recorded from seven consecutive crank cycles while fifteen recreational cyclists (11 males, 4 females; age: 25.8 ± 4.5 years; height: 1.7 ± 0.1 m; mass: 67.2 ± 9.8kg; mean ± one standard deviation) rode a stationary bicycle at 30 rpm with a power output at ~30 W, representing a typical application of ergometer cycling in a rehabilitation setting [30]. Participants had signed a prior written consent form approved by the Institutional review board at the University of Massachusetts Amherst (Protocol #: 2017–4085). Muscle activations were determined by electromyography (EMG) from tensor fasciae latae (TFL), rectus femoris (RF), biceps femoris long head (BFL), semitendinosus (ST), vastus lateralis (VL), vastus medialis (VM), lateral gastrocnemius (LGA), medial gastrocnemius (MGA), soleus (SOL), and tibialis anterior (TA). For each muscle EMG amplitude was normalized by the average of its peak EMG values across the seven crank cycles. Crank torque was computed by multiplying the crank arm length (0.17m) by the tangential pedal force over the crank cycle. The central difference method was used to compute crank angular velocity and acceleration using the filtered crank angle data [31]. Anterior and upward pedal forces and counterclockwise crank and pedal angles were defined as positive values. The pedal angle and pedal force data for the right leg were generated by assuming symmetry and shifting the left leg data by 180° of the pedal cycle. Mean pedal angles and pedal forces averaged across participants were used as data tracking targets for the pedaling simulations. Mean crank angular velocity from the participants was 188°/s (31 rpm), matching closely with the target cadence of 30 rpm. Further details on the collection and processing of the experimental data are described in an earlier publication [29].

To scale the model segments, lower body segment lengths were measured in a standing posture for each participant and averaged to compute mean anthropometric data. To determine the initial model posture, the pelvic tilt angle was measured by markers placed on the pelvis and recorded via still photos with the participants seated on the bicycle in a pedaling posture with the left pedal at the top dead center position. The mean pelvic angle was tilted 11.5° anteriorly, while the model pedal angle was adjusted by 12.5° to align it with the experimental data.

### Dynamic tracking optimization

Tracking optimizations were performed to simulate a two-legged pedaling motion over one complete crank cycle that matched kinematic and kinetic data from human participants using the DC method [13,21]. The freely available optimization solver IPOPT [19] was used to solve the optimization problem in MATLAB using the MEX function interface [13].

The objective function included both tracking and activation terms (Eq 1), similar to that used to generate simulations of human walking [14,32]. The first term (tracking) represents how well the model reproduces experimental pedal angles and forces [33]. The second term (activation) is the sum of cubed MTU activation integrals, known to produce distributed muscle activations that minimize muscle fatigue [16,34–36]. The objective function, *J*, is given by

$$J=W_{1}\left(\frac{\sum_{i=1}^{v}\sum_{j=1}^{n}{\left(\frac{Y_{ij}-{\hat{Y}}_{ij}}{{SD}_{ij}}\right)}^{2}}{v\times n}\right)+W_{2}\left(\frac{\sum_{m=1}^{nMus}\int_{o}^{t}a_{m}^{3}(t)}{nMus\times n}\right)$$ (2)

where Y_ij_ are experimental pedal angle, and horizontal and vertical pedal force components, Ŷ_ij_ are the same variables from the model simulated pedaling motion, SD_ij_ are inter-subject standard deviations, ***v*** is the number of variables being compared (6 variables), *a_m_* is the activation of the m^th^ muscle at a given time, ***nMus*** is the number of muscles (18 muscles), ***n*** is the number of data points in a complete crank cycle, **W**_**1**_ and **W**_**2**_ are weighting values for the tracking and activation terms, respectively. Letters *i*, *j*, and *m* are indices for ***v***, ***n***, and ***nMus*** respectively. A sensitivity analysis was performed to determine how the weighting between tracking and activation terms affected the quality of the optimal solution (see following section).

The states in the model were hip, knee, and ankle joint angles and angular velocities, crank angle and angular velocity, muscle activations, and muscle CC lengths, leading to a total of 50 states ([7 angles × 2] + [18 muscles × 2]). The controls were the 18 MTU excitation signals. For the DC method, the time series of states and controls were discretized into equally-spaced nodes (***n***), leading to a total of 68 × ***n*** unknowns.

The system dynamical equations were discretized into ***n*** equally-spaced nodes, converting the system equations into algebraic equality constraints using the backward Euler method.

$$\frac{x_{i+1}-x_{i}}{\Delta t}-f_{i+1}=0,i=1,2,\ldots ,n-1$$ (3)

where *Δt* is the difference between *t*_*i+1*_ and *t*_*i*_ and ***f***_*i+1*_ represents the time derivatives of the states ***x(t)*** at time *i+*1 (50 states × (***n***-1)). The states and controls at the first node were assumed to be the same as the sates and controls at the final node, except for the crank angle, which was fixed at 0° for the first node and 360° for the final node. OpenSim does not provide the dynamic equations of motion in symbolic form, so the sparsity pattern of the constraints Jacobian matrix, which is required for the IPOPT optimization algorithm, was generated manually. The OpenSim function ‘*computeStateVariableDerivatives*’ was used to calculate the state variable time derivatives at each node. The time to complete one crank cycle (*tFinal*) was set at 1.914s, the mean duration observed in the experiment data. Thus, the total number of equality constraints varied from 569 and 9069 based on the selected value used for ***n*** ranging from 11 to 181 (number of constraints = 50 × (***n***−1) + 68 + 1, ***n*** = 11, 31, 51, 91 or 181).

The DC approach generally converges better if the initial guess is already dynamically consistent [13]. Initial guesses for the states and controls were produced by generating multiple forward simulations using the OpenSim forward dynamics tool, with manual muscle excitation patterns based on published pedaling simulation and EMG studies [5,36–38]. The states and controls from this forward simulation will be referred to as the dynamic initial guess. We also used an initial guess where the states and controls were the same for all nodes (i.e., no movement, constant activation), referred to as the static initial guess. All optimizations were performed on the same laptop computer with a 2.7 GHz Intel i7-6820HQ CPU and 16 GB of RAM, running OpenSim 3.3, MATLAB R2018a, and IPOPT release 3.11.0.

### Sensitivity analyses

We performed three sensitivity analyses to determine how different weightings, node densities, and initial conditions affected the optimal solutions. First, to examine which combination of objective function weights W_1_ and W_2_ could predict physiologically realistic MTU excitation patterns with low tracking errors, six combinations of W_1_ and W_2_ were used to solve the optimization problem at 31 equally-spaced nodes with the dynamic initial guess. The six pairs included ‘tracking only’ (W_1_ = 1, W_2_ = 0) and ‘activation only’ (W_1_ = 0, W_2_ = 1) solutions. Between these two extremes, the tracking term weight was held at W_1_ = 1, with the activation term weight varied from 0.5 to 10 (W_2_ = 0.5, 1, 5, 10). Secondly, to examine the effect of node spacing, tracking optimizations with equal weightings (W_1_ = W_2_ = 1) were solved at node densities from 11 to 181 (***n*** = 11, 31, 51, 91, and 181) with the dynamic initial guess. The relationship between the node number and CPU time was evaluated by the correlation coefficient (‘*corrcoef*’, MATLAB). The number of nodes represent the discretization of the states and controls in the solution process, corresponding to *Δt* ranging between 0.1914 s and 0.0106 s. Finally, the effect of the dynamic versus static initial guess was evaluated by solving the tracking optimization using each initial guess with the equal weightings (W_1_ = W_2_ = 1) at 31 equally-spaced nodes.

The quality of optimal solutions was evaluated by comparing the objective function tracking term values, with smaller values indicating better matching of pedal angles and pedal forces between the simulated and experimental data. Simulated MTU excitation patterns were qualitatively evaluated by visual comparison with burst timings and shapes in corresponding experimental EMG patterns from six muscles (RF, BFL, VL, LGA, SOL, and TA). Further, the tracking quality was considered to be acceptable when simulated pedal angles and pedal forces were fell within one standard deviation of the experimental data, which is a commonly used criterion in evaluating musculoskeletal simulation results [6,8].

## Results

### Effects of different objective term weightings

**Fig 2:**
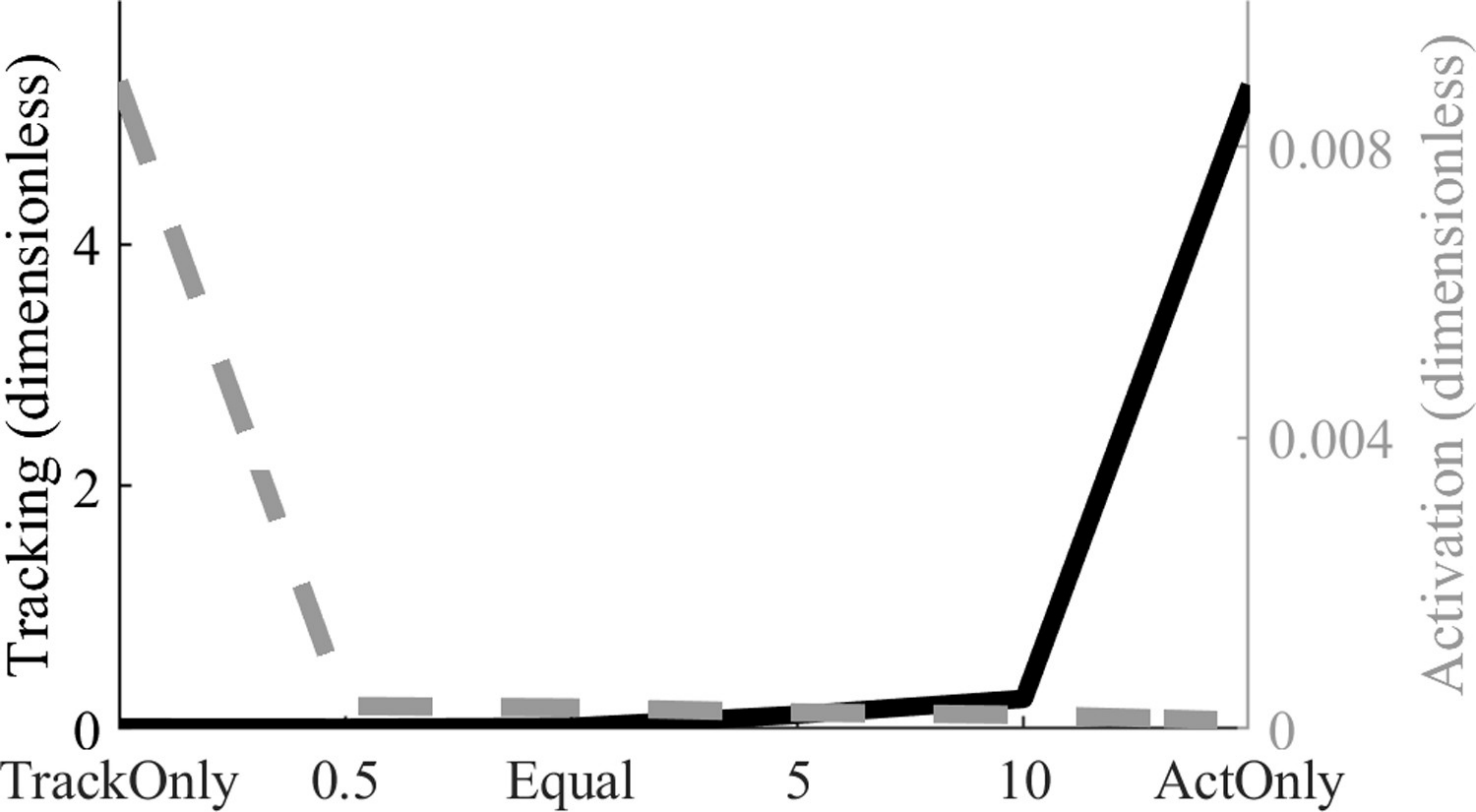
The effects of changing relative weightings in the objective function tracking and activation terms. Objective function values for the optimizations with six combinations of tracking and activation term weightings. ‘TrackOnly’ weighting was 1 for tracking and 0 for activation (W_1_ = 1, W_2_ = 0); ‘ActOnly’ weighting was 0 for tracking and 1 for activation (W_1_ = 0, W_2_ = 1). In four other simulations the activation term weight was .5, 1 (‘Equal’), 5 or 10 while the tracking weight was fixed at 1. Smaller tracking term values indicate better match between the simulated and experimental data. Note that high tracking quality could be maintained with low muscle activation when the activation term weight was 0.5 or 1.

The first sensitivity analysis examined the effects of different objective function term weightings on the quality of the optimal solutions at 31 nodes. The tracking term represents the ability of the simulated pedal angles and pedal forces to match the corresponding experimental data, while the activation term is the sum of cubed MTU activation integrals. There were trade-offs associated with weighing one term much more heavily than the other (Fig 2). With tracking and activation weights set at 0 and 1 respectively (W_1_ = 0, W_2_ = 1, ActOnly), the objective function value due to activation was small (0.0001) but the value due to tracking had its highest value (5.3080), representing the greatest tracking error. In the opposite case (W_1_ = 1, W_2_ = 0, TrackOnly), the objective function value due to tracking was greatly reduced to 0.0105 representing excellent fit with the experimental data, but the activation term was increased to its largest value (0.0089).

**Fig 3:**
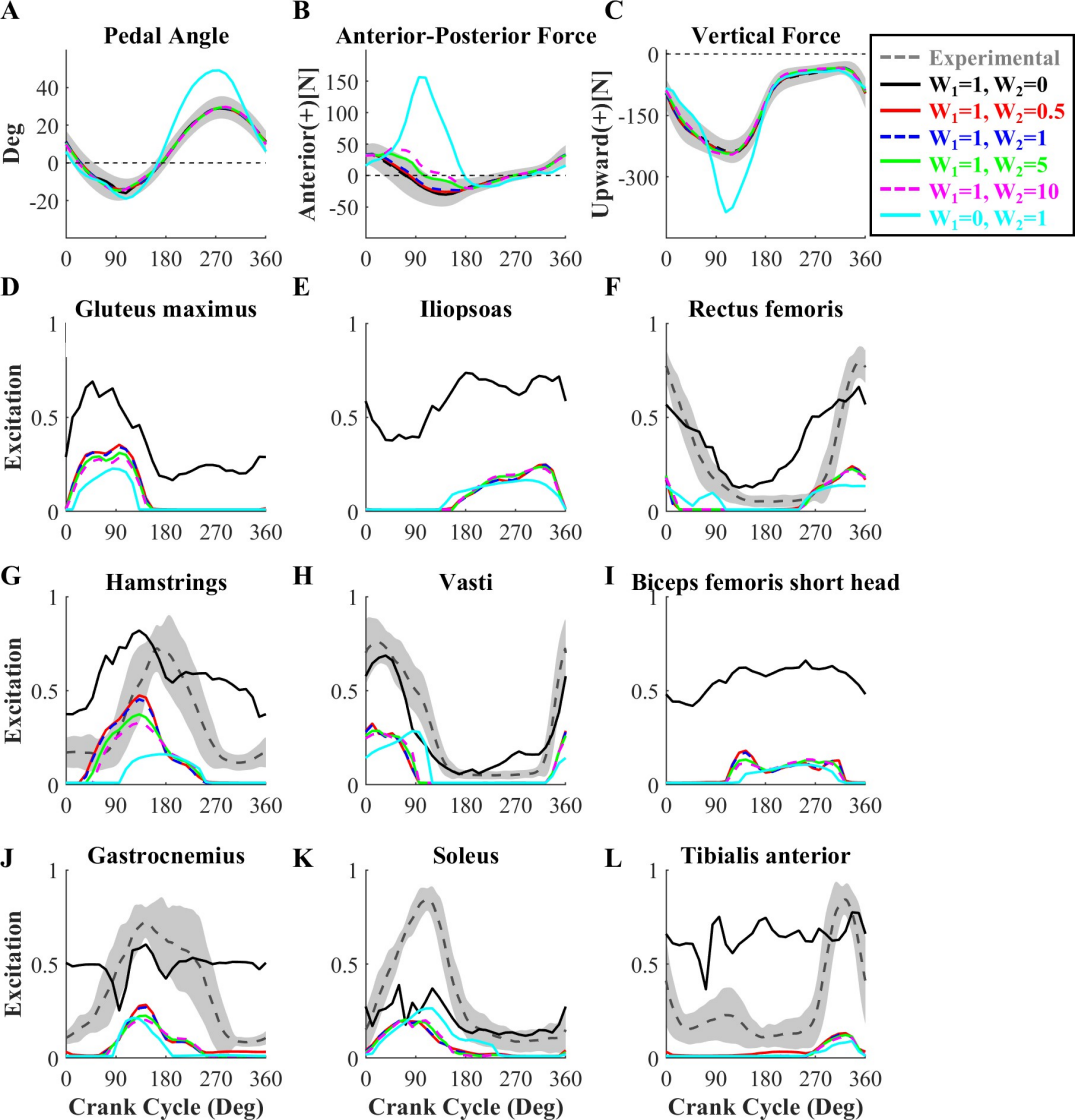
Simulated pedal angles, pedal forces, and MTU excitation patterns using different objective function term weightings. Simulated patterns of pedal angle (A), pedal forces (B and C), and MTU excitations (D-L) from simulations using the six combinations of objective term weightings compared with experimental data (*dark gray solid line*, *with gray shaded area representing* ±*1 SD*). Because peak timings throughout the seven crank cycles were varied, the maximum values in the averaged experimental EMG patterns was below one. The tracking term weight was held at W_1_ = 1, while the activation term weight (W_2_) was set to 0 (TrackOnly, *black solid line*), 0.5 (*red solid line*), 1 (*blue dotted line*), 5 (*green solid line*), and 10 (*magenta dotted line*). In ActOnly, the tracking term weight was W_1_ = 0 and the activation term weight was W_2_ = 1 (*cyan solid line*). Experimental EMG data for the gluteus maximus, iliopsoas, and biceps femoris short head were not available. Simulated data from the left leg are shown, those from the contralateral leg are almost identical. Either 0.5 or 1 weighting for the tracking term produced high tracking quality with physiologically realistic muscle activation patterns.

The optimal solutions for activation term weights of 0.5 and 1 were successful in terms of tracking, with the model simulated pedal angles and pedal forces tracking the experimental data closely, well within ±1 standard deviation (Fig 3A–3C). MTU excitation patterns were also well matched with experimental EMG data with these weightings (Fig 3F–3H and 3J–3L), although the onset timing of HAM was slightly earlier than observed in the BFL EMG pattern. As expected the TrackOnly weightings also resulted in model pedal angles and pedal forces that fell within ±1 standard deviation (Fig 3A–3C). However, unrealistically high model MTU activations over much of the crank cycle were found (Fig 3D–3L), with the gastrocnemius and tibialis anterior MTU excitation patterns not phasic as observed in the EMG data (Fig 3J and 3L). On the contrary, for the ActOnly condition the MTU activation timing was generally matched with burst timings and shapes observed in the experimental EMG data; however, the model pedal angle and pedal force data deviated substantially from the experimental (Fig 3A–3C). When the activation term weighting was increased to 5 or 10, the model anterior-posterior pedal force became less accurate during the downstroke (around 90°, Fig 3B), perhaps associated with less HAM activity compared to the simulations with term weights of 0.5 or 1. Thus, either the half (0.5) or equal (1) weighting for the activation term could attain both high tracking quality and physiologically realistic muscle activation patterns.

### Effects of the number of nodes in the discretization

**Fig 4:**
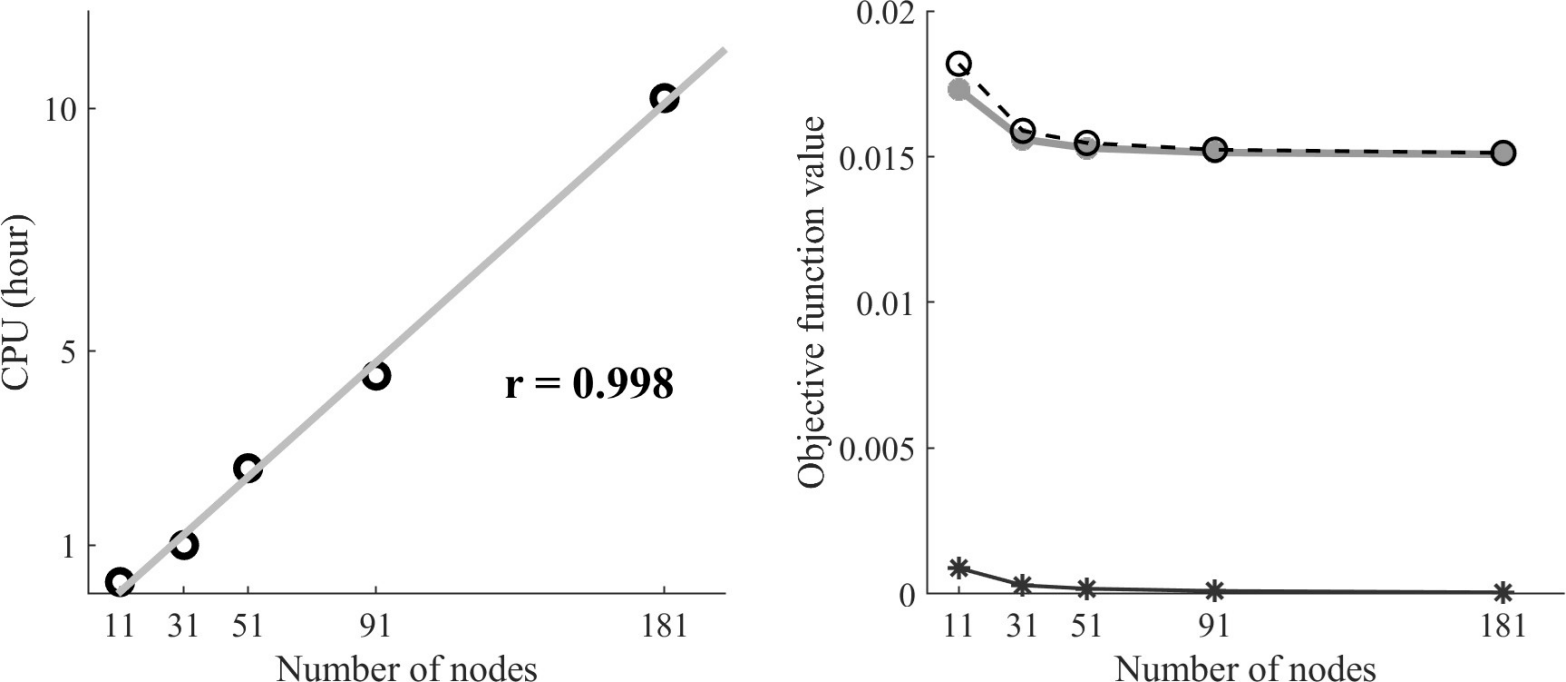
Effects of changing the number of nodes in the discretization on the CPU time and objective function value. (A) The CPU time increased almost linearly as a function of the number of nodes. (B) The total objective function value (*black dashed line*) decreased 12.7% from 11 to 31 nodes, with little further change above 31 nodes. The slight changes in the total objective function value were reflected in both the activation term (*dark gray solid line)* and the tracking term (*light gray solid line*). Note that the tracking term can in principle go to zero (i.e. perfect tracking), while the activation term cannot.

**Fig 5:**
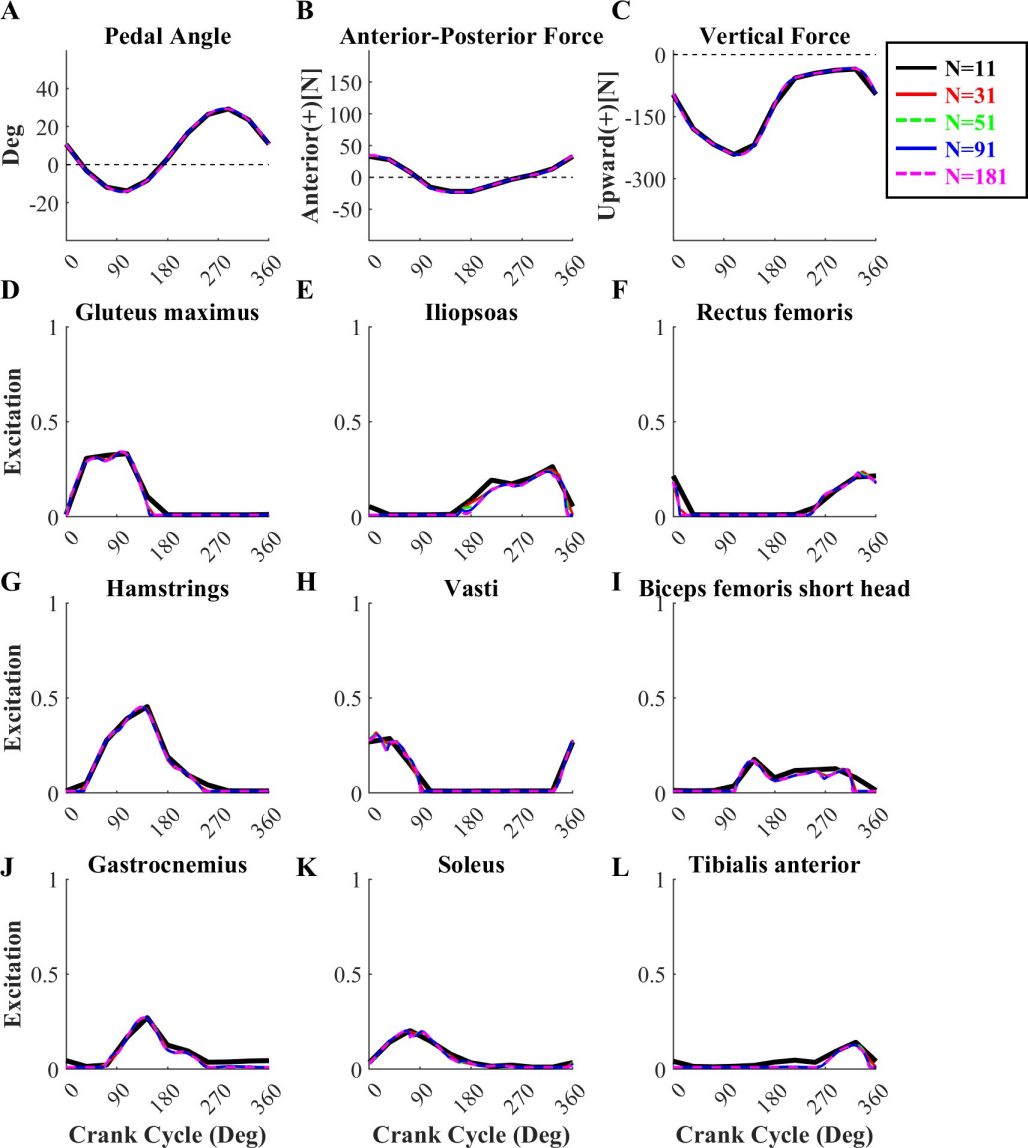
Simulated pedal angles, pedal forces, and MTU excitation patterns at each node. Simulated pedal angles (A), pedal forces (B-C), and MTU excitation patterns (D-L) obtained for simulations using 11 (*black solid line*), 31 (*red solid line*), 51 (*green dotted line*), 91 (*blue solid line*), and 181 (*magenta dotted line*) nodes compared with experimental pedal angles and forces (*dark gray dotted line*, *with gray shaded area representing* ±*1 SD*). The optimal solution quality was similar for temporal node densities of 31 or greater.

When solving the tracking optimization with both objective function term weights set to 1, the computational cost increased almost linearly as the number of nodes increased from 11 to 181 (r = 0.998, p < 0.001). Although the optimization took only 24 minutes to solve with 11 nodes (Fig 4A), the burst timings and shapes in MTU excitation patterns deviated from those found in the optimal solutions with the other higher node numbers due to insufficient node density (Fig 5D–5L). When the node number was changed from 11 to 31, the objective function value dropped significantly (12.7%), resulting in nearly identical simulated model pedal angles, pedal forces, and MTU excitation patterns to those found in the optimal solutions with the higher node numbers (Fig 5). The dynamic optimization took 1 hour (60.2 minutes) to solve with 31 nodes, increasing to 10.2 hours with 181 nodes (Fig 4A). However, the increased cost did not result in a substantially better solution, as the objective function decreased by only a small amount with the higher node density (Fig 4B). The total objective function did decrease slightly (1.9%) from 31 to 51 nodes, due to decreases in both tracking and activation term values. However, the use of node densities above 31 had almost no effect on the simulated model pedal angles, pedal forces or MTU excitation patterns (Fig 5).

### Effect of different initial guesses

**Fig 6:**
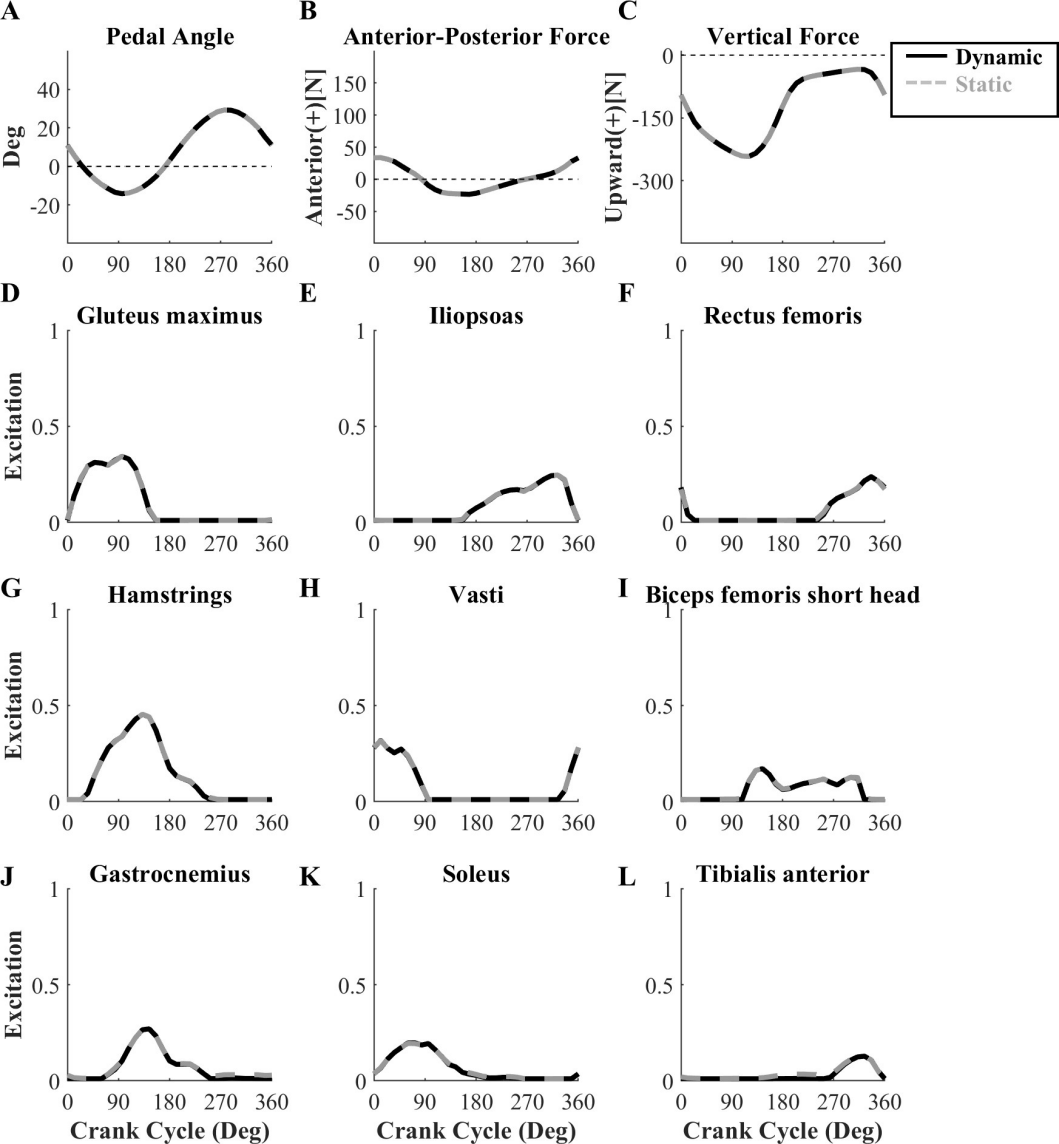
Simulated pedal angles, pedal forces, and MTU excitation patterns using two different initial guesses. Simulated pedal angles (A), pedal forces (B-C), and MTU excitation patterns (D-L) found with dynamic (*black solid line*) and static (*light gray dotted line*) initial guesses. Initial states and controls were generated from OpenSim forward simulations with manually input MTU excitation patterns for the dynamic initial guess, but were the same for all nodes (i.e. no movement, constant activation) for the static initial guess. The simulated results for the two initial guesses were almost identical, but the optimized solution started from the dynamic initial guess was obtained in less time.

Simulated pedal angles, pedal forces, and MTU excitation patterns estimated with the static initial guess were almost identical to those predicted with the dynamic initial guess (Fig 6). In the final objective function values, the activation term value was 0.0003 for both guesses, and the tracking term value was 0.0156 for the dynamic initial guess and 0.0158 for the static initial guess. However, the optimization started from the static initial guess took ~1.6 times longer to converge (97.3 minutes *versus* 60.2 minutes for the dynamic guess).

## Discussion

We have presented an approach for generating optimal control simulations of seated pedaling using the DC method within the OpenSim and MATLAB environments. The optimal control pedaling problem was solved with different weights on the terms in the objective function, different numbers of temporal nodes, and two different types of initial guesses to test how those factors affected tracking quality and predicted MTU excitation patterns. The relative weighting of the objective function terms had a substantial impact on the solution quality and must be selected judiciously. On the other hand, the data tracking quality and MTU excitation patterns were relatively insensitive to the temporal node densities over the range considered despite wide variation in computational cost. The quality of the optimal solution was also not affected by the type of initial guess, but the computational cost was greater with the static initial guess compared with the dynamically consistent guess.

It is common to use hybrid objective functions that combine data tracking and muscular effort terms, and in previous DC studies that approach has resulted in realistic kinematics, kinetics, and MTU excitation patterns [14,15,32]. However, the weighting between the tracking and effort terms should be selected carefully. We identified a range of weights (Fig 2) that led to similarly good simulation results (Fig 3). However, outside of that range there were either discrepancies in the kinematic and kinetic data, or excessive and sometimes non-phasic MTU excitations, either of which would negatively impact interpretations based on the simulation results. The relative weights for the tracking and effort terms in prior studies were different from those found here [14,15,32], and likely cannot be generalized across studies. The ideal weights will depend on several factors such as model, the task being simulated, and the specific quantities being tracked. Thus, the fact that equal weighting of the tracking and effort terms was found to yield good results is specific to the problem considered here and should be closely evaluated if the model is used for simulating other pedaling tasks or if different formulations of the objective terms are used.

The current runtimes of 24 minutes to 10 hours on a commodity laptop computer across 11 to 181 temporal nodes were longer than the 20 minutes to 3 hours across 21 to 240 nodes reported in an earlier study where a similar pedaling optimization problem was solved [15]. In the current study, the optimal solution quality began to degrade on coarse grids such as 11 nodes (*Δt* = 0.191 s), which is consistent with the previous finding of degraded solution quality below 50 nodes in simulating half gait cycle (*Δt* = 0.013 s) [39]. While it is difficult to compare across modeling studies due to the varied time resolutions originating from the interaction between simulated time and node density [12,14,15], the greater computational costs in our study are likely due to overhead associated with frequent calls from MATLAB to external OpenSim functions (e.g., calculating state derivatives, spring forces), and the use of finite differences for derivatives. In the earlier DC pedaling study [15], analytical derivatives were used in solving the optimal control problem, which can provide a substantial computational performance advantage. Analytical derivatives are not currently possible within OpenSim, though the potential advantage of using algorithmic differentiation with OpenSim has recently been demonstrated [40]. While there is some overhead associated with using finite differences, OpenSim provides an easy to use environment for musculoskeletal modeling with a robust API that facilitates implementation of the DC method [13]. Moreover, the quality of solutions obtained with the OpenSim-MATLAB interface on relatively coarse temporal grids were similar to the results on finer grids, and can be obtained in much less time than is required for the more common direct shooting methods that have been used for simulating pedaling [6,15,41].

The results obtained with the static and dynamic initial guesses were identical; however, it took ~1.6 times longer to solve the problem using the static initial guess compared with the dynamic initial guess, which is consistent with previous findings. In a prior study using the same algorithm, optimizations beginning from a static initial guess, as defined in the current study, took as much as 4 times longer compared with optimization solved as part of a grid refinement approach, where the initial guess is the optimal solution to the same problem obtained on a coarser temporal grid [13]. Likewise, a jumping optimization problem started from an initial guess produced by the computed muscle control algorithm solved approximately 3 times faster than did same problem starting from an initial guess based on non-optimal bang-bang controls [12]. Better convergence starting from an initial guess closer to the optimal solution is not surprising, but another important consideration with the DC method is whether the initial guess is dynamically consistent. Using an initial guess that already satisfies the system dynamical equations, even if it is far from the optimal solution, is not required for the DC method, but can lead to faster convergence.

We initially modeled the interaction between the rider and bicycle as a closed kinematic loop, as commonly reported in past research (e.g., 6,41) using the “PointConstraint” feature in OpenSim. While kinematic constraints can be included in OpenSim models, in our pilot work we found that it was not possible to use models with kinematic constraints with our DC method implemented from within MATLAB. Therefore, we modeled the foot-pedal interaction with a stiff (100,000 N/m) spring using the “PointToPointSpring” force element in OpenSim. This approach considers that the interaction between the foot and the pedal is not perfectly rigid and provides an easy means to extract pedal forces during simulations. The pedal forces obtained with this approach were in good agreement with experimental data (Fig 5). While the spring-based foot-pedal constraint worked well in practice, the newly released OpenSim Moco optimal control framework can accommodate kinematic constraints and thus would allow the same pedaling problems to be solved using a closed kinematic loop if desired [42].

Aside from comparing simulated muscle activations with EMG data there are few opportunities to assess the other muscle-level variables in the model. The MTU CC lengths can be compared with ultrasound-based fascicle length measurements, though few such data are available for pedaling. The fascicle length of vastus lateralis was reported in an experimental study to change from 0.091 to 0.127 m in pedaling as the knee joint flexed [43], which was similar to the VAS MTU CC length change in our model (0.079 to 0.123 m). Gastrocnemius muscle fascicle length was found to change between 0.040 to 0.055 m in walking [44] while we found GAS MTU CC length to change from 0.028 to 0.0423 m in the model. These shorter lengths for the gastrocnemius CC make sense due to the much more flexed knee joint in pedaling than in walking [22,45,46]. More broadly, MTU CC length trajectories over the crank cycle across all muscles also remained within a physiologically reasonable range (S1 Fig). Specifically, fascicle length changes of all the MTUs were between the lower and upper bounds of the force-length curves. Together, these results suggest that the observed MTU CC lengths during simulated pedaling were physiologically reasonable. Future studies using ultrasound measurement across a greater range of muscles during pedaling can aid to verify the interpretation [43,44].

### Study limitations

The computational performance results presented here are specific to the particular model used and pedaling problem that was solved and may not generalize to other scenarios. The bicycle-rider model developed in this study had eighteen MTUs with a constrained seated posture and motion only permitted in the sagittal plane, combined with models of similar complexity having been used to successfully simulate pedaling in earlier studies (e.g., 5,6,8). The trade-off between the temporal discretization and computational cost may be different than those reported here if the model or task are changed, such as simulating non-seated cycling or including 3-D pelvis motion.

Bilateral symmetry for the experimental pedal angle and pedal forces with a 180° phase offset was assumed, which has been a common assumption in earlier pedaling simulation studies [6,8]. Pedaling in able-bodied people is generally symmetrical in leg muscle activity [47] though small asymmetries have been documented in specific variables [48–50], and larger asymmetries can be expected in certain clinical populations who are affected unilaterally, such as in stroke [51] or unilateral amputation [52]. While bilateral symmetry was assumed in this study it is not a requirement of the approach described here, which could also be used to study asymmetrical pedaling in future studies.

The generation of the sparsity pattern for the constraints Jacobian matrix is challenging and may be prone to errors. We produced the constraints Jacobian matrix manually because the system equations of motion were not available in a symbolic form from OpenSim. Failing to identify all of the non-zero elements in the Jacobian matrix will prevent finding the optimal result, while using a dense Jacobian dramatically increases computational costs [13]. We have provided the constraints Jacobian sparsity patterns for the cases included in this study, but applying to approach to new problems will require generating the Jacobian patterns from scratch. A useful hybrid approach is adopted in the new OpenSim Moco software to automatically generate the sparsity pattern, at the expense of treating some sparse blocks as dense and therefore not achieving the maximal possible computational performance.

### Summary

The optimal control pedaling problem was successfully solved using the DC approach via an OpenSim-MATLAB interface. This flexible and computationally efficient framework should facilitate the use of optimal control methods to study the biomechanics, energetics, and control of human pedaling. Interested readers may freely access the model and example code to use and modify for their own research at the GitHub repository (http://github.com/sangsoopark1739/pedaling_DC_OpenSim).

## Supporting information

S1 Fig Contractile component (CC) length changes over the crank cycle. Those CC length changes originated from the optimal solution with 31 nodes and equal weightings. All MTU CC length changes were fell between the lower and upper bound of their force-length curve, suggesting that the CC length changes estimated from the optimization are physiologically relevant.
(TIF)

S1 File General overview: Development of a bicycle-rider model. This overview is about the description for the model development process.
(DOCX)

## References

[1] Pandy MG. Computer modeling and simulation of human movement. Annu Rev Biomed Eng. 2001;3(1):245–73. doi: 10.1146/annurev.bioeng.3.1.245 11447064

[2] Umberger BR, Caldwell GE. Musculoskeletal Modealing. In: Robertson DGE, Caldwell GE, Hamill J, Kamen G& Whittlesey SN(Eds.), Research methods in biomechanics. 2nd ed. Champaign, IL: Human Kinetics; 2014. 247–276 p.

[3] Umberger BR, Miller RH. Optimal Control Modeling of Human Movement. In: Müller B. et al. (eds) Handbook of Human Motion. Berlin: Springer; 2017. 1–22 p.

[4] Valero-Cuevas FJ, Hoffmann H, Kurse MU, Kutch JJ, Theodorou EA. Computational models for neuromuscular function. IEEE Rev Biomed Eng. 2009;2:110–35. doi: 10.1109/RBME.2009.2034981 21687779 PMC3116649

[5] Raasch CC, Zajac FE, Ma B, Levine WS. Muscle coordination of maximum-speed pedaling. J Biomech. 1997;30(96):595–602. doi: 10.1016/s0021-9290(96)00188-1 9165393

[6] Neptune RR, Hull ML. Evaluation of performance criteria for simulation of submaximal steady-state cycling using a forward dynamic model. J Biomech Eng. 1998;120(3):334–41. doi: 10.1115/1.2797999 10412400

[7] Ting LH, Raasch CC, Brown D a, Kautz SA, Zajac FE. Sensorimotor state of the contralateral leg affects ipsilateral muscle coordination of pedaling. J Neurophysiol. 1998;80(3):1341–51. doi: 10.1152/jn.1998.80.3.1341 9744943

[8] Gidley AD, Marsh AP, Umberger BR. Performance criteria for generating predictive optimal control simulations of bicycle pedaling. Comput Methods Biomech Biomed Engin. 2019;22(1):11–20. doi: 10.1080/10255842.2018.1522535 30398070

[9] Schutte LM, Rodgers MM, Zajac FE, Glaser RM. Improving the Efficacy of Electrical Stimulation- Induced Leg Cycle Ergometry: An Analysis Based on a Dynamic Musculoskeletal Model. IEEE Trans Rehabil Eng. 1993;1(2):109–25.

[10] van Soest AJ, Casius LJ. Which factors determine the optimal pedaling rate in sprint cycling? Med Sci Sports Exerc. 2000;32(11):1927–34. doi: 10.1097/00005768-200011000-00017 11079524

[11] Zajac FE, Neptune RR, Kautz S a. Biomechanics and muscle coordination of human walking: Part I: Introduction to concepts, power transfer, dynamics and simulations. Gait Posture. 2002;16:215–32. doi: 10.1016/s0966-6362(02)00068-1 12443946

[12] Porsa S, Lin Y-C, Pandy MG. Direct Methods for Predicting Movement Biomechanics Based Upon Optimal Control Theory with Implementation in OpenSim. Ann Biomed Eng. 2015;. doi: 10.1007/s10439-015-1538-6 26715209

[13] Lee L-F, Umberger BR. Generating optimal control simulations of musculoskeletal movement using OpenSim and MATLAB. PeerJ. 2016;4:e1638. doi: 10.7717/peerj.1638 26835184 PMC4734202

[14] Lin Y-C, Pandy MG. Three-dimensional data-tracking dynamic optimization simulations of human locomotion generated by direct collocation. J Biomech. 2017;59:1–8. doi: 10.1016/j.jbiomech.2017.04.038 28583674

[15] Kaplan ML, Heegaard JH. Predictive algorithms for neuromuscular control of human locomotion. J Biomech. 2001;34(8):1077–83. doi: 10.1016/s0021-9290(01)00057-4 11448699

[16] Ackermann M, van den Bogert AJ. Optimality principles for model-based prediction of human gait. J Biomech. 2010;43(6):1055–60. doi: 10.1016/j.jbiomech.2009.12.012 20074736 PMC2849893

[17] Delp SL, Anderson FC, Arnold AS, Loan P, Habib A, John CT, et al. OpenSim: open-source software to create and analyze dynamic simulations of movement. IEEE Trans Biomed Eng. 2007;54(11):1940–50. doi: 10.1109/TBME.2007.901024 18018689

[18] Seth A, Hicks JL, Uchida TK, Habib A, Dembia CL, Dunne JJ, et al. OpenSim: Simulating musculoskeletal dynamics and neuromuscular control to study human and animal movement. PLoS Comput Biol. 2018;14(7). doi: 10.1371/journal.pcbi.1006223 30048444 PMC6061994

[19] Wächter A, Biegler LT. On the implementation of an interior-point filter line-search algorithm for large-scale nonlinear programming. Math Program. 2006;106(1):25–57.

[20] Nguyen VQ, Johnson RT, Sup FC, Umberger BR. Bilevel optimization for cost function determination in dynamic simulation of human gait. IEEE Trans Neural Syst Rehabil Eng. 2019;27(7):1426–35. doi: 10.1109/TNSRE.2019.2922942 31199264

[21] Betts JT. Practical methods for optimal control and estimation using nonlinear programming. SIAM; 2010.

[22] Lai AKM, Arnold AS, Wakeling JM. Why are Antagonist Muscles Co-activated in My Simulation? A Musculoskeletal Model for Analysing Human Locomotor Tasks. Ann Biomed Eng. 2017;45(12):2762–74. doi: 10.1007/s10439-017-1920-7 28900782 PMC5989715

[23] Yamaguchi GT, Zajac FE. A planar model of the knee joint to characterize the knee extensor mechanism. J Biomech. 1989;22(1):1–10. doi: 10.1016/0021-9290(89)90179-6 2914967

[24] Yamaguchi GT. Dynamic modeling of musculoskeletal motion: a vectorized approach for biomechanical analysis in three dimensions. Springer Science & Business Media; 2001.

[25] Millard M, Uchida T, Seth A, Delp SL. Flexing computational muscle: modeling and simulation of musculotendon dynamics. J Biomech Eng. 2013;135(2):21005. doi: 10.1115/1.4023390 23445050 PMC3705831

[26] Anderson DE, Madigan ML, Nussbaum MA. Maximum voluntary joint torque as a function of joint angle and angular velocity: model development and application to the lower limb. J Biomech. 2007;40(14):3105–13. doi: 10.1016/j.jbiomech.2007.03.022 17485097 PMC6820133

[27] Riener R, Edrich T. Identification of passive elastic joint moments in the lower extremities. J Biomech. 1999;32(5):539–44. doi: 10.1016/s0021-9290(99)00009-3 10327008

[28] Fregly BJ, Zajac FE, Dairaghi CA. Bicycle drive system dynamics: theory and experimental validation. J Biomech Eng. 2000;122(4):446–52. doi: 10.1115/1.1286678 11036570

[29] Park S, Caldwell GE. Muscular activity patterns in 1-legged vs. 2-legged pedaling. J Sport Heal Sci [Internet]. 2021;10(1):99–106. Available from: https://www.sciencedirect.com/science/article/pii/S2095254620300107. doi: 10.1016/j.jshs.2020.01.003 33518019 PMC7858030

[30] Ambrosini E, De Marchis C, Pedrocchi A, Ferrigno G, Monticone M, Schmid M, et al. Neuro-mechanics of recumbent leg cycling in post-acute stroke patients. Ann Biomed Eng. 2016;44(11):3238–51. doi: 10.1007/s10439-016-1660-0 27251336 PMC5093201

[31] Winter DA. Biomechanics and Motor Control of Human Movement. 4th ed. Hoboken, New Jersey: John Wiley & Sons; 2009.

[32] van den Bogert AJ, Blana D, Heinrich D. Implicit methods for efficient musculoskeletal simulation and optimal control. Procedia IUTAM. 2011;2:297–316. doi: 10.1016/j.piutam.2011.04.027 22102983 PMC3217276

[33] Neptune RR, Kautz SA, Zajac FE. Muscle contributions to specific biomechanical functions do not change in forward versus backward pedaling. J Biomech. 2000;33(2):155–64. doi: 10.1016/s0021-9290(99)00150-5 10653028

[34] Anderson FC, Pandy MG. Static and dynamic optimization solutions for gait are practically equivalent. J Biomech. 2001;34(2):153–61. doi: 10.1016/s0021-9290(00)00155-x 11165278

[35] Crowninshield RD, Brand RA. A physiologically based criterion of muscle force prediction in locomotion. J Biomech. 1981;14(11):793–801. doi: 10.1016/0021-9290(81)90035-x 7334039

[36] Thelen DG, Anderson FC, Delp SL. Generating dynamic simulations of movement using computed muscle control. J Biomech. 2003;36:321–8. doi: 10.1016/s0021-9290(02)00432-3 12594980

[37] Hug F, Boumier F, Dorel S. Altered muscle coordination when pedaling with independent cranks. Front Physiol. 2013;4:232. doi: 10.3389/fphys.2013.00232 24009587 PMC3755179

[38] Neptune RR, Herzog W. Adaptation of muscle coordination to altered task mechanics during steady-state cycling. J Biomech. 2000;33(2):165–72. doi: 10.1016/s0021-9290(99)00149-9 10653029

[39] van den Bogert AJ, Hupperets M, Schlarb H, Krabbe B. Predictive musculoskeletal simulation using optimal control: effects of added limb mass on energy cost and kinematics of walking and running. Spec Issue Artic Proc IMechE Part P J Sport Eng Technol. 2012;226(2):123–33.

[40] Falisse A, Serrancolí G, Dembia CL, Gillis J, De Groote F. Algorithmic differentiation improves the computational efficiency of OpenSim-based trajectory optimization of human movement. PLoS One. 2019;14(10):e0217730. doi: 10.1371/journal.pone.0217730 31622352 PMC6797126

[41] Umberger BR, Gerritsen KGM, Martin PE. Muscle fiber type effects on energetically optimal cadences in cycling. J Biomech. 2006;39(8):1472–9. doi: 10.1016/j.jbiomech.2005.03.025 15923008

[42] Dembia CL, Bianco NA, Falisse A, Hicks JL, Delp SL. OpenSim Moco: musculoskeletal optimal control. PLOS Comput Biol. 2020;16(12):e1008493. doi: 10.1371/journal.pcbi.1008493 33370252 PMC7793308

[43] Muraoka T, Kawakami Y, Tachi M, Fukunaga T. Muscle fiber and tendon length changes in the human vastus lateralis during slow pedaling. J Appl Physiol. 2001;91(5):2035–40. doi: 10.1152/jappl.2001.91.5.2035 11641341

[44] Lichtwark GA, Bougoulias K, Wilson AM. Muscle fascicle and series elastic element length changes along the length of the human gastrocnemius during walking and running. J Biomech. 2007;40(1):157–64. doi: 10.1016/j.jbiomech.2005.10.035 16364330

[45] Chapman A, Vicenzino B, Blanch P, Hodges P. Do differences in muscle recruitment between novice and elite cyclists reflect different movement patterns or less skilled muscle recruitment? J Sci Med Sport. 2009;12(1):31–4. doi: 10.1016/j.jsams.2007.08.012 18077215

[46] Winter DA. Kinematic and kinetic patterns in human gait: variability and compensating effects. Hum Mov Sci. 1984;3(1–2):51–76.

[47] Carpes FP, Diefenthaeler F, Bini RR, Stefanyshyn DJ, Faria IE, Mota CB. Influence of leg preference on bilateral muscle activation during cycling. J Sports Sci. 2011;29(2):151–9. doi: 10.1080/02640414.2010.526625 21120741

[48] Carpes FP. Bilateral pedaling asymmetry during a simulated 40… 2007;. 17369798

[49] Daly DJ, Cavanagh PR. Asymmetry in bicycle ergometer pedalling. Med Sci Sports. 1976;8(3):204–8. doi: 10.1249/00005768-197600830-00013 979569

[50] Hunt MA, Sanderson DJ, Moffet H, Inglis JT. Interlimb asymmetry in persons with and without an anterior cruciate ligament deficiency during stationary cycling. Arch Phys Med Rehabil. 2004;85(9):1475–8. doi: 10.1016/j.apmr.2003.10.017 15375819

[51] Chen H-Y, Chen S-C, Chen J-JJ, Fu L-L, Wang YL. Kinesiological and kinematical analysis for stroke subjects with asymmetrical cycling movement patterns. J Electromyogr Kinesiol. 2005;15(6):587–95. doi: 10.1016/j.jelekin.2005.06.001 16051498

[52] Childers WL, Kistenberg RS, Gregor RJ. Pedaling asymmetries in cyclists with unilateral transtibial amputation: Effect of prosthetic foot stiffness. J Appl Biomech. 2011;27(4):314–21. doi: 10.1123/jab.27.4.314 21896953

