# Supplementary figures and images for: A direct collocation framework for optimal control simulation of pedaling using OpenSim
Source: PLoS One. 2022 Feb 22;17(2):e0264346. doi: 10.1371/journal.pone.0264346 (PMC8863267; doi:10.1371/journal.pone.0264346)

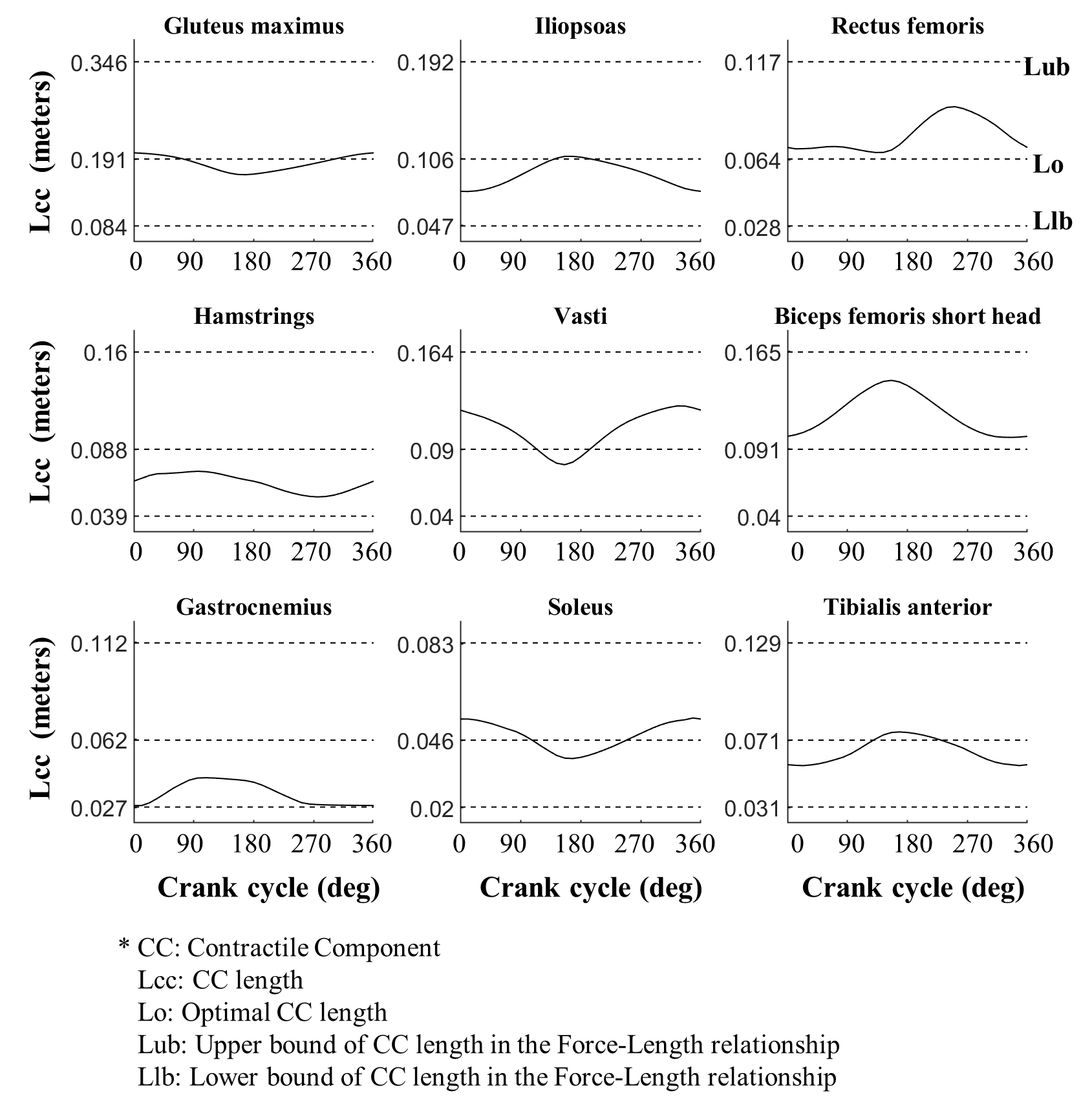

Supplement: S1 Fig — Those CC length changes originated from the optimal solution with 31 nodes and equal weightings. All MTU CC length changes were fell between the lower and upper bound of their force-length curve, suggesting that the CC length changes estimated from the optimization are physiologically relevant. (TIF) [file pone.0264346.s001.tif]
