# Supplementary material for: A direct collocation framework for optimal control simulation of pedaling using OpenSim
Source: PLoS One. 2022 Feb 22;17(2):e0264346. doi: 10.1371/journal.pone.0264346 (PMC8863267; doi:10.1371/journal.pone.0264346)
Supplement: S1 File — This overview is about the description for the model development process. (DOCX) [file pone.0264346.s002.docx]

**Supplementary material to accompany: A direct collocation framework for optimal control simulation of pedaling using OpenSim**

Sangsoo Park^1,2^, Graham E. Caldwell^1^, and Brian Umberger^3^

^1^ Department of Kinesiology, University of Massachusetts Amherst, Amherst MA

^2^ College of Medicine, Korea University, Seoul, South Korea

^3^ School of Kinesiology, University of Michigan, Ann Arbor, MI

**General overview: development of a bicycle-rider model**

In OpenSim, we developed a two dimensional (2-D) two-legged bicycle-rider model (Fig. S1) that represents mean anthropometric characteristics and force-producing ability of fifteen human participants [1,2]. First, we created a 2-D lower body model consisting of eight rigid segments and nine muscle tendon units (MTUs) per leg, based on a recent model (Fig. S1) developed to simulate movements with large leg flexion angles such as pedaling [3]. To better match force-producing ability and anthropometric characteristics of the human participants, we selected fiber length and tendon slack length of each MTU within physiological boundaries and then scaled the model with experimental anthropometric measures. Further, we selected maximum isometric force of each MTU and changed parameters that determine the force-extension curve of the passive elastic component (PEC) to better represent maximum isometric joint torque and passive joint torque production ability of the human participants. Initial pedal posture was chosen by a constrained optimization using mean experimental pedal angle data from the participants. Pedal-foot interaction was modeled by a spring with high stiffness, creating a closed kinematic loop with a ‘soft’ constraint at the pedal-foot interface. More details about the muscle model parameter selection and the process to determine the initial pedal posture follow.


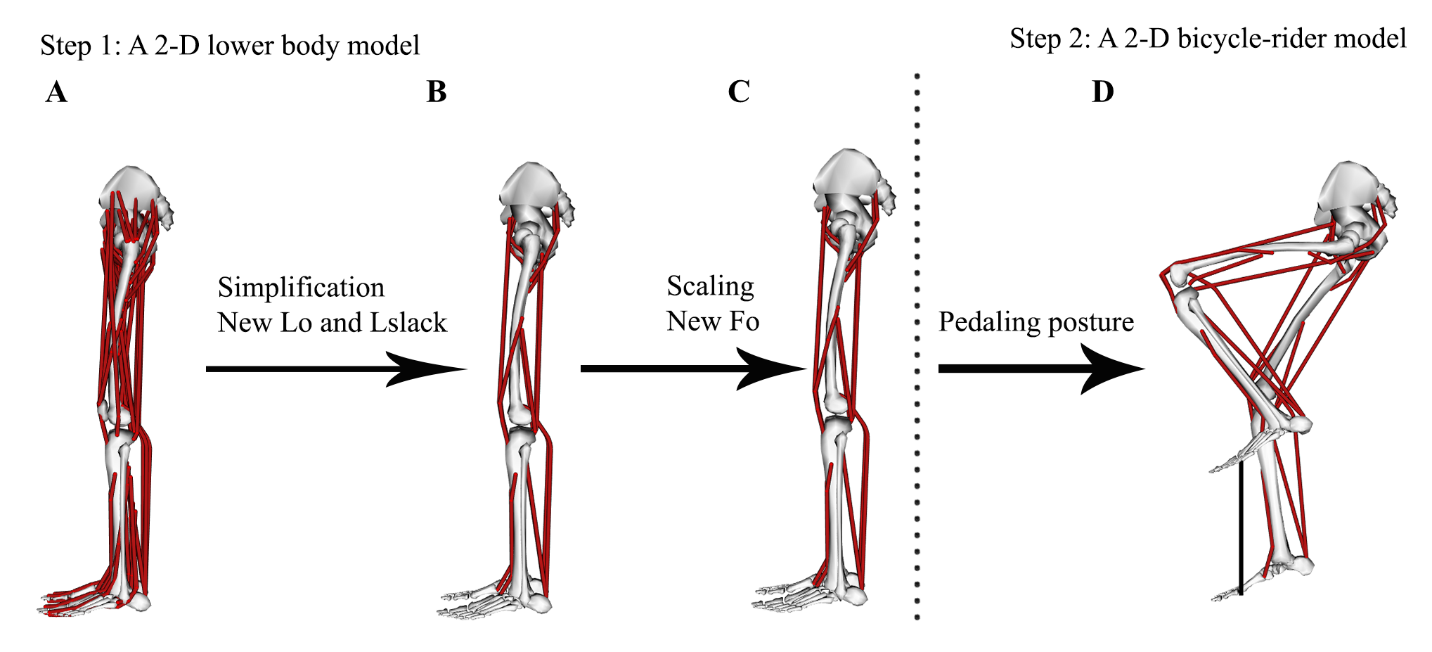


**Fig S1. A procedure to develop a 2-D two-legged bicycle-rider model.**

(A) Lai’s model in OpenSim (B) A 2D lower body model with new Lo and Lslack values with nine MTUs per leg (C) A 2-D model with new Fo values and mean anthropometric characteristics of the human participants (D) Initial posture of the 2-D model forming the closed kinematic chain.

**A generic 2-D lower body model**

A generic 2-D lower body musculoskeletal model was created in OpenSim [1], based upon a recent model developed to simulate movements with large leg flexion angles such as pedaling [3]. Our modified lower body model contained eight rigid segments that represent the bicycle crank, the pelvis, and paired thighs, lower legs, feet (including massless pedals). The body segments articulated via hinge joint to form the hip, knee, and ankle joints, and the hip joint and the rotation axis of the crank were fixed in space. The combined rotational inertia of the crank arms, pedals, chainrings, and the rear wheel was represented by a single inertia value representing a 42/28 gear ratio [4]. Morphology and inertial properties of each segment originated from an available musculoskeletal model in OpenSim with segment dimensions based on an average-sized (1.7 m tall, 75 kg) adult male [3].

Each leg was actuated by nine muscle tendon units (MTUs): iliopsoas (IP), gluteus maximus (GMAX), vasti (VAS), rectus femoris (RF), hamstrings (HAM), biceps femoris short head (BFsh), gastrocnemius (GAS), soleus (SOL), and tibialis anterior (TA). This grouping of synergistic muscles is similar in complexity to other musculoskeletal models used to accurately simulate 2D pedaling movement [5,6]. The anatomical path and pennation angle of each MTU was determined from muscles with the largest force capability among muscle synergists with similar anatomical function [3]. Due to computational difficulties associated with including the patella segment in our pilot work, we replaced the patella with moving path points that reproduce the VAS and RF anatomical paths as a function of knee joint angle. Moment arm lengths as a function of knee joint angle from our new VAS and RF paths were consistent with the Lai et al. (2017) model for both muscles [3]. Millard’s three-component equilibrium muscle model was used to represent each MTU, due to its computational efficiency and ability to match experimental data [7]. Each MTU force producing ability was determined by force-velocity-length (F-V-L) relationships of contractile component (CC), and force-extension (F-Ext) relationships of series elastic (SEC) and parallel elastic (PEC) components. The default parameters were used to define the scaled CC F-V-L and SEC F-Ext relationships, which were same for all MTUs. Neuromuscular activation and deactivation times were set to 10 ms and 40 ms for all the MTUs, based on previous work reporting 5 to 20 ms for activation and 20 to 60 ms for deactivation for leg muscles (Millard et al., 2013; Winters & Stark, 1988).

**Muscle parameter selection and scaling**

The remaining unknown muscle parameters were Lo and Fo of CC and Lslack of SEC for the nine MTUs. Initially, parameters were drawn from the Lai study [3] to simulate two-legged pedaling motion. However, initial simulations produced large excitation of GAS during the upstroke, which is inconsistent with medial gastrocnemius muscle EMG data in pedaling [9]. This anomaly in GAS activity could originate from high passive net joint torque production at the knee joint in the upstroke during which the knee extensors are overstretched. Additionally, we found co-activity of all flexion related muscles during the upstroke due to both insufficient maximum hip flexion torque and excessive passive hip extensor torque. To reduce these anomalous effects, we altered muscle parameters and scaled the model segments to obtain realistic maximum active isometric and passive net joint torque values as observed in human participants.

We first estimated physiologically realistic maximum active isometric and passive net joint torque values from literature reports. Maximum flexion/extension net joint torque values for the hip, knee, and ankle were estimated by regression equations developed from dynamometer measurements on young healthy adults [10]. In pedaling, the largest hip and knee extensor torques occur during the downstroke with the hip (~90°) and knee (~110°) joints highly flexed [11,12]. The ankle joint angle varied from -20° (flexion) to 10° (extension) in pedaling [11]. The regression equations were specific for participant characteristics such as sex, height, and mass. In the experimental pedaling data [9], there were 11 males (mean 1.75 m tall, 71.4 kg) and 4 females (1.6 m, 55.7 kg). Based on those observations, we computed maximum flexor and extensor net joint torque values separately for males and females as the hip flexed from -20° to -100°, the knee flexed from 40° to 120°, and the ankle extended from -20° to 40°. The mean characteristics for each sex were combined in a weighted average to represent the joint torque capabilities of the experimental participants. In addition, we estimated passive net joint torque values in 10° increments with double-exponential equations developed from experimental data of healthy male adults [13]. Passive net hip joint torque was computed from 40° to 100° with the knee flexed at 10° and the ankle at 0°. Passive net knee joint torque was computed from 60° to 120° with the hip at 70° and the ankle at 0°. Passive ankle joint torque was computed from -20° to 30° with the hip and knee flexed at 80°. These were considered as experimental passive net joint torque values.

Based on these experimental active and passive net joint torque values, we performed a sequential process to give the model realistic torque production ability. First we selected Lo and Lslack values within one standard deviation of the values reported in a previous study [14], producing muscle parameter values within a physiological range [3]. A 2D generic model was updated with those new Lo and Lslack values (Table S1), followed by scaling with experimental anthropometric measures to reflect participant segment lengths. Further, Fo of each MTU was chosen (Table S1) to better match with the mean experimental torque data, as in other modeling studies on pedaling [6,15]. The model with the new Lo, Lslack, and Fo values had greater maximum net joint torque values in general (Fig S2), suggesting the model is stronger than the mean of the human participants.

**Table S1.** Muscle model parameter values selected for this study (SEL) compared with those reported in a previous study by Rajagopal [14].

|  | Lo (cm) | | Lslack (cm) | | Fo (N) | | |
| --- | --- | --- | --- | --- | --- | --- | --- |
|  | Rajagopal | SEL | Rajagopal | SEL | Rajagopal | SEL |  |
| HAM | 6.9 ± 1.8 | 8.7 | 34.8 ± 2.1 | 36.8 | 2201 ± 645 | 3201 |  |
| BFsh | 11.0 ± 2.1 | 8.9 | 10.6 ± 2.6 | 8.0 | 557 ± 158 | 1073 |  |
| GMAX | 15.7 ± 2.6 | 18.3 | 6.8 ± 4.4 | 11.2 | 1406 ± 260 | 3338 |  |
| PSOAS | 11.7 ± 1.7 | 10.0 | 10.0 ± 2.2 | 11.0 | 1427 ± 306 | 2448 |  |
| RF | 7.6 ± 1.3 | 6.3 | 44.8 ± 1.4 | 43.4 | 2192 ± 473 | 2192 |  |
| VAS | 9.9 ± 1.8 | 8.8 | 22.1 ± 1.9 | 24.0 | 5149 ± 1025 | 5149 |  |
| GAS | 5.1 ± 1.0 | 5.9 | 39.9 ± 1.1 | 40.0 | 3116 ± 727 | 2300 |  |
| SOL | 4.4 ± 1.0 | 4.4 | 27.7 ± 1.0 | 28.1 | 6195 ± 1606 | 4195 |  |
| TA | 6.8 ± 0.8 | 6.8 | 24.0 ± 1.0 | 24.1 | 1227 ± 205 | 1027 |  |


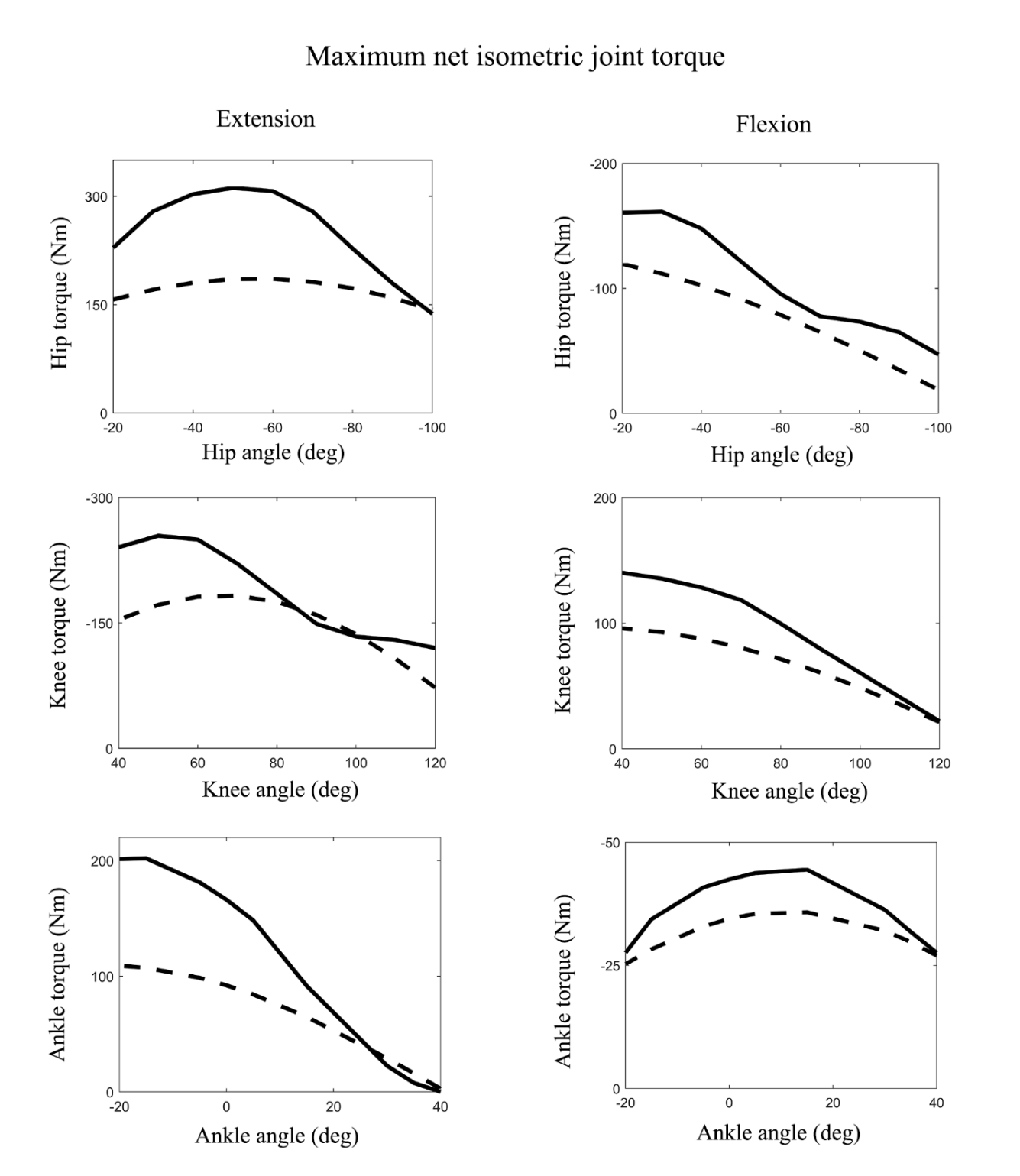


**Fig S2. Comparison of maximum net joint torque values.**

Net joint torques from our musculoskeletal model (*black solid line*) compared with experimental isometric joint torque data (*black dashed line*) from the literature [10].

We manually adjusted the default parameters that define the PEC F-Ext relation for HAM, BFsh, GMAX, RF, VAS, and TA to better match the model and experimental passive joint torques (Table S2). The model produced smaller passive joint torques but similar patterns compared to human participants (Fig S3). The experimental passive joint torques originate from elasticity within various anatomical structures such as joint capsules, ligaments, and parallel connective tissues within muscle and tendon. In contrast, the MTU model parallel elastic component represents elasticity of structures parallel to the CC and SEC only. Because model passive joint torques were produced by the MTU PECs only, it is not surprising that they are smaller than the experimental values.

**Table S2**. Comparison of the default parameters for PEC F-Ext relation of each MTU with those selected for model (SEL); changed parameter values are in ***bold Italic***.

|  | Default parameters | | | | | SEL parameters | | | | |
| --- | --- | --- | --- | --- | --- | --- | --- | --- | --- | --- |
|  | S_ZF | S_ONF | LF | SF_ONF | CV | S_ZF | S_ONF | LF | SF_ONF | CV |
| HAM | 0 | 0.7 | 0.2 | 2.86 | 0.75 | 0 | 0.7 | ***0.1*** | 2.86 | 0.75 |
| BFsh | 0 | 0.7 | 0.2 | 2.86 | 0.75 | 0 | ***1.4*** | ***0.01*** | ***1.429*** | ***1*** |
| GMAX | 0 | 0.7 | 0.2 | 2.86 | 0.75 | 0 | 0.7 | 0.2 | 2.86 | 0.75 |
| PSOAS | 0 | 0.7 | 0.2 | 2.86 | 0.75 | 0 | 0.7 | 0.2 | 2.86 | 0.75 |
| RF | 0 | 0.7 | 0.2 | 2.86 | 0.75 | 0 | ***1.4*** | ***0.05*** | ***1.429*** | ***1*** |
| VAS | 0 | 0.7 | 0.2 | 2.86 | 0.75 | 0 | ***1.4*** | ***0.05*** | ***1.429*** | ***1*** |
| GAS | 0 | 0.7 | 0.2 | 2.86 | 0.75 | 0 | 0.7 | 0.2 | 2.86 | 0.75 |
| SOL | 0 | 0.7 | 0.2 | 2.86 | 0.75 | 0 | ***1.4*** | 0.2 | ***1.429*** | 0.75 |
| TA | 0 | 0.7 | 0.2 | 2.86 | 0.75 | 0 | ***1.4*** | ***0.1*** | ***1.429*** | ***1*** |

* Abbreviations: S_ZF for strainAtZeroForce, ST_ONF for strainAtOneNormForce, LF for stiffnessAtLowForce, SF_ONF for stiffnessAtOneNormForce, and CV for Curviness


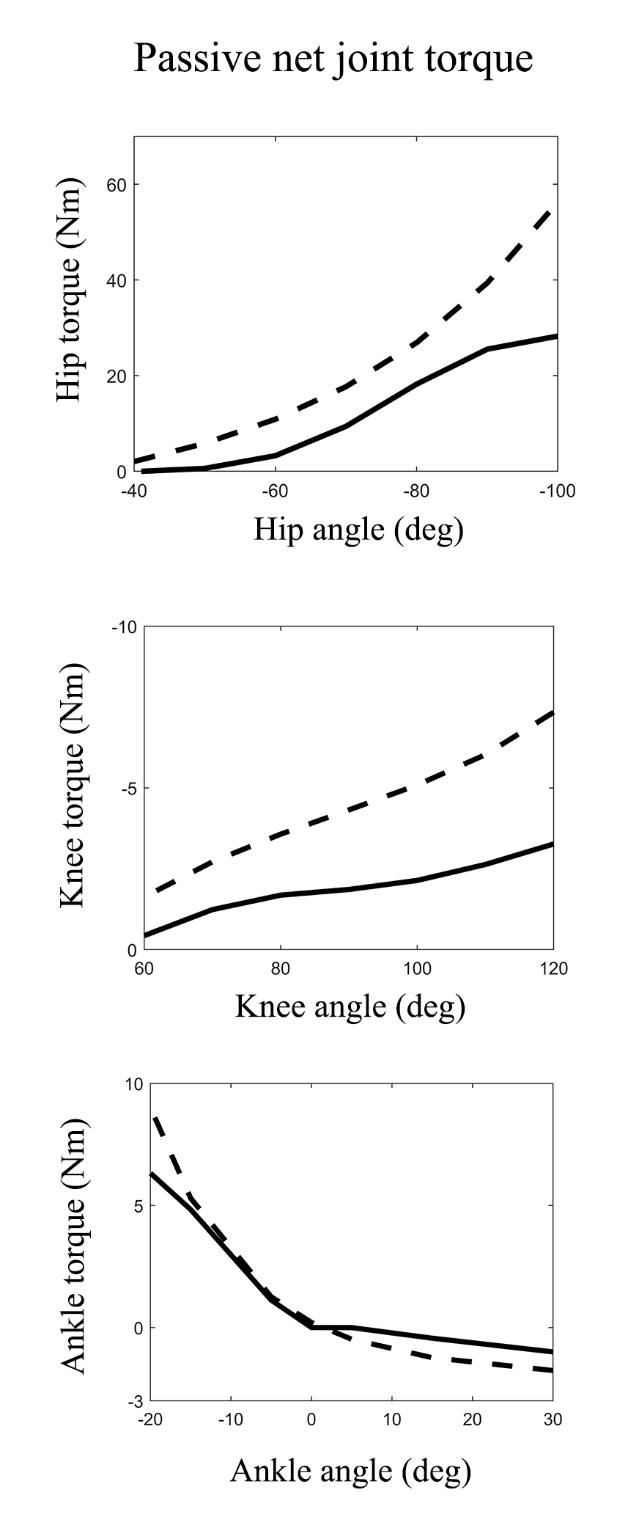


**Fig S3. Comparison of passive joint torque values**

Passive net joint torques from our musculoskeletal model (*black solid line*) compared with experimental passive joint torque data (*black dashed line*) from the literature [13].

**Initial posture and crank position to model the foot-pedal interface**

An important design consideration was the nature of the foot-pedal interface. Very stiff springs (k=100,000) were used (‘*PointToPointSpring*’ function in OpenSim) to connect the metatarsalphalangeal (MTP) joint to the ends of the crank, creating a closed kinematic chain with the body segments and crank arms. For accurate placement of the foot/pedal interface springs, an initial pedal posture and position of the crank needed to be determined. As experimental joint angle data were not available, an optimization procedure was used to set the initial posture of the model and crank position.

The remaining joint positions for the initial posture were determined from an optimization procedure with experimental data from three crank arm locations (0°, 180°, and 360°). The eleven optimization design variables included three joint angles in the left leg at the three crank locations (3x3=9) and the location of the crank center displacement relative to the pelvis center. The optimization algorithm sought to minimize a two-part objective function:

${J= W}_{s1}\left( \frac{\sum_{i=1}^{3} {({Model\_PedalAngle}_{i}-{Exp\_PedalAngle}_{i})}^{2}}{N} \right)+W_{s2}\left( \frac{\sum_{i=1}^{3} {( \vec{{MTP}_{i}}- \vec{{CRANK}_{i}})}^{2}}{N} \right) (S1)$

where *i* represents the crank angles, MTP is a 2D vector representing A/P and vertical location of the left MTP joint, and CRANK is a 2D vector indicating A/P and vertical location of the end of the crank arm. The objective function relative weights W_s1_ and W_s2_ were set at one and five, respectively, to place greater emphasis on the foot/pedal spring location. The left and right leg joint angles were assumed to be 180° out-of-phase. The optimal solution was used to define the initial posture of the lower body model when the crank angle is at 0°, followed by placing the stiff springs (spring constant, k=100,000) that connect the MTP joints to the ends of the crank arms.

**References**

1. Delp SL, Anderson FC, Arnold AS, Loan P, Habib A, John CT, et al. OpenSim: open-source software to create and analyze dynamic simulations of movement. IEEE Trans Biomed Eng. 2007;54(11):1940–50.

2. Seth A, Hicks JL, Uchida TK, Habib A, Dembia CL, Dunne JJ, et al. OpenSim: Simulating musculoskeletal dynamics and neuromuscular control to study human and animal movement. PLoS Comput Biol. 2018;14(7).

3. Lai AKM, Arnold AS, Wakeling JM. Why are Antagonist Muscles Co-activated in My Simulation? A Musculoskeletal Model for Analysing Human Locomotor Tasks. Ann Biomed Eng. 2017;45(12):2762–74.

4. Fregly BJ, Zajac FE, Dairaghi CA. Bicycle drive system dynamics: theory and experimental validation. J Biomech Eng. 2000;122(4):446–52.

5. Neptune RR, Kautz SA, Zajac FE. Muscle contributions to specific biomechanical functions do not change in forward versus backward pedaling. J Biomech. 2000;33(2):155–64.

6. Raasch CC, Zajac FE, Ma B, Levine WS. Muscle coordination of maximum-speed pedaling. J Biomech. 1997;30(96):595–602.

7. Millard M, Uchida T, Seth A, Delp SL. Flexing computational muscle: modeling and simulation of musculotendon dynamics. J Biomech Eng. 2013;135(2):21005.

8. Winters JM, Stark L. Estimated mechanical properties of synergistic muscles involved in movements of a variety of human joints. J Biomech. 1988;21(12):1027–41.

9. Park S, Caldwell GE. Muscular activity patterns in one-legged versus two-legged pedaling. J Sport Heal Sci. 2020;

10. Anderson DE, Madigan ML, Nussbaum MA. Maximum voluntary joint torque as a function of joint angle and angular velocity: model development and application to the lower limb. J Biomech. 2007;40(14):3105–13.

11. Chapman A, Vicenzino B, Blanch P, Hodges P. Do differences in muscle recruitment between novice and elite cyclists reflect different movement patterns or less skilled muscle recruitment? J Sci Med Sport. 2009;12(1):31–4.

12. Gregor RJ, Cavanagh PR, LaFortune M. Knee flexor moments during propulsion in cycling—A creative solution to Lombard’s Paradox. J Biomech. 1985;18(5):307–16.

13. Riener R, Edrich T. Identification of passive elastic joint moments in the lower extremities. J Biomech. 1999;32(5):539–44.

14. Rajagopal A, Dembia CL, DeMers MS, Delp DD, Hicks JL, Delp SL. Full-body musculoskeletal model for muscle-driven simulation of human gait. IEEE Trans Biomed Eng. 2016;63(10):2068–79.

15. Neptune RR, Hull ML. Evaluation of performance criteria for simulation of submaximal steady-state cycling using a forward dynamic model. J Biomech Eng. 1998;120(3):334–41.
